# Deep-Learning-Based Parking Area and Collision Risk Area Detection Using AVM in Autonomous Parking Situation

**DOI:** 10.3390/s22051986

**Published:** 2022-03-03

**Authors:** Sunwoo Lee, Dongkyu Lee, Seok-Cheol Kee

**Affiliations:** 1 Department of Smart Car Engineering, Chungbuk National University, 1 Chungdae-ro, Seowon-gu, Cheongjusi 28644, Korea; dltjsdn3606@chungbuk.ac.kr (S.L.); dlehdrb3909@chungbuk.ac.kr (D.L.); 2 Department of Intelligent Systems and Robotics, Chungbuk National University, 1 Chungdae-ro, Seowon-gu, Cheongjusi 28644, Korea

**Keywords:** autonomous driving, parking area segmentation, collision risk area, image recognition, deep learning

## Abstract

In this paper, I propose a bird eye view image detection method for parking areas and collision risk areas at the same time in parking situations. Deep learning algorithms using area detection and semantic segmentation were used. The main architecture of the method described in this paper is based on a harmonic densely connected network and a cross-stage partial network. The dataset used for training was calibrated to four 190° wide-angle cameras to generate around view monitor (AVM) images based on the Chungbuk National University parking lot, and an experiment was performed based on this dataset. In the experimental results, the available parking area was visualized by detecting the parking line, parking area, and available driving area in the AVM images. Furthermore, the undetected area in the semantic segmentation as a collision risk area was visualized in order to obtain the results. According to the proposed attention CSPHarDNet model, the experimental results were 81.89% mIoU and 18.36 FPS in a NVIDIA Xavier environment. The results of this experiment demonstrated that algorithms can be used in real time in a parking situation and have better performance results compared to the conventional HarDNet.

## 1. Introduction

### 1.1. Research Background

For a long time, camera-based object recognition technology has been studied through digital image processing. Recently, with the advent and development of deep learning and neural networks, there have been tremendous developments in the field of computer vision. Among them, camera-based object recognition technology is an important research field for autonomous driving; therefore, the autonomous driving field has also experienced much development. With the development of computer vision based on deep learning, many algorithms for detecting moving objects, such as pedestrians and vehicles, and stationary objects, such as signs and obstacles, have emerged. Object classification is the most basic field and involves algorithms that classify objects by recognizing them in images. Object detection involves algorithms that detect objects in an image or video using a bounding box. Object segmentation involves algorithms that group together similar parts of an image that belong to the same class. In addition, there is image captioning, which describes an image as text, and object tracking, which tracks an image. In this way, studies on image-based cognitive algorithms have progressed significantly since deep learning, and research on object detection, in particular, has shown the greatest development in the field of autonomous driving.

In this study, in an autonomous parking scenario, four 190° vehicle cameras were calibrated, and, using an AVM-based deep learning algorithm, the parking area was investigated via the detection of parking lines, as well as collisions with other fixed objects, such as walls and parked cars. Moreover, a study was conducted to detect the risk area through the semantic segmentation method.

In parking lot data-based semantic segmentation labeling, mainly used in this study, if RGB is set on the object to be detected, ground truth is created, deep-learning-based learning is performed, and segmented inference results can be created. Thereafter, RGB-based object detection can be performed through image processing. For the system configuration for semantic segmentation, a harmonic densely connected network (HarDNet) [1] structure was used to compensate for the lack of real-time performance due to the excessive amount of DenseNet computation [2]. A cross-stage partial network [3] was used to reduce the amount of computation. In addition, attention was added to each block to improve the overall image segmentation and performance for each class. The parking area and collision risk area—detected through semantic segmentation—were visualized in AVM using OpenCV.

Network learning was performed by adjusting the loss function, optimization, and data augmentation, and the experimental results were obtained through experiments on indoor and outdoor environments and an integrated experiment based on the best-performing learning technology. For network optimization, I learned that, by using AMP and using ONNX-based TensorRT for Jetson Xavier, I could improve the frames per second (FPS) by more than twofold.

### 1.2. Research Purpose

Many studies have been conducted that propose algorithms to improve on previous studies to detect parking areas in autonomous parking. In a previous study, an algorithm was used to find a parking area using object detection and to distinguish whether or not parking was possible using object classification for the found area. Most representatively, like VPS-Net [4], a previous study detected all parking surfaces using object detection, and had a classification structure that was able to distinguish available and impossible parking surfaces from among detected parking surfaces. The main proposals of our study are listed below:

- Unlike the object detection method, based on the semantic segmentation algorithm with a simple structure, the algorithm proposed in this paper can simultaneously detect the drivable area, parking area, and parking line to essentially classify the parking and non-parking areas. It is an algorithm that can even detect areas with potential collision risks, such as walls, columns, parked vehicles, and pedestrians;
- In this study, a simpler model was investigated in an end-to-end method using semantic segmentation without using various algorithms. In addition, a study was conducted to detect not only the parking space but also the collision risk area by using the undetected part after detecting the parking space, parking line, stop bar, and drivable space;
- This study also includes an experiment to improve the performance through various techniques with the same architecture in the process of area detection for autonomous parking. First, experiments were conducted through four types of data augmentation to improve the performance due to the lack of training data. Then, the experiment was conducted while adjusting the loss function and optimizer, and the loss function and optimizer that yielded the best results were used. In addition, optimization studies capable of reducing the memory and learning speed during the learning process in order to infer faster inferences after learning is completed were also conducted.

## 2. Related Work

### 2.1. Segmentation Networks

Semantic segmentation is largely classified into the encoder and decoder. Similar to classification, the encoder detects a feature map through convolution and classifies the input image; the decoder plays a role in restoring the class-classified feature map to the size of the original input image. In this study, the encoder creates a network structure using DenseNet, and the decoder recovers the size of the input image through bilinear interpolation. Interestingly, the semantic segmentation method was commercialized with the advent of FCN [5], which changed the fully connected layer to 1 × 1 convolution layers for class division using a CNN-based VGGNet [6] network. Net [7] and PSPNet [8], with an improved performance, have emerged, and use feature maps of various scales, with the pyramid pooling module and ICNet [9] using various image scales.

In this paper, the proposed—implemented—network is based on DenseNet, HarDNet, and CSPNet. DenseNet is a structure that connects the output feature map from the previous layer through the input and channel sum of the next layer, so that the feature maps of each layer are densely connected. Since it does not use many channels like ResNet, it offers a good performance with a minimum number of channels. The most significant feature of DenseNet is that it connects the feature maps of all layers and has a structure that connects the feature maps of the previous layer to the feature maps of all subsequent layers. This structure solves two problems. First, most CNN models that appeared before DenseNet had a problem in that the weight of the feature map was not transmitted to the next feature map as the layer deepened. DenseNet solved this problem with a structure in which all layers are connected. Moreover, since the initial value is transmitted to the last layer, the gradient loss problem is also alleviated.

Another characteristic of DenseNet is that the numbers of parameters and computations are minute. DenseNet uses a small number of channels since all the layers are connected; therefore, using a large number of channels will result in extensive computation. HarDNet is a structure proposed by DenseNet and has fewer channels and parameters. The implementation speed is slow and difficult to use. Therefore, in this study, HarDNet was used. In HarDNet, some of the feature maps of each layer are connected and the size of the channel is formed in a harmonic manner. The most essential part of the HarDNet structure can be found in the output of the harmonic-type block structure.

In an even-numbered layer of blocks, the channel size is given to k (growth rate) to increase the size of the channel, and a constant channel size is maintained in the odd-numbered layers. In the output of one block, only the channels of the feature maps of odd-numbered layers are summed and passed on to the next layer. Here, the feature maps of even-numbered layers with a large size do not enter the output because of the weight.

As neural networks develop, studies that prove that the performance improves as they become deeper and wider have emerged, and researchers have studied deep and wide architectures accordingly. As a result, researchers began attentively studying how to reduce the amount of computation, and the advent of CSPNet made it possible to reduce the weight of many algorithms. CSPNet has a simple structure. In DenseNet-based CSPNet, only half of the channel of the input feature map is calculated through the DenseNet block; the other half is transferred to the transition layer by summing the channels with the output feature map of the block.

Considering CSPNet intuitively, it is understandable that the amount of computation is reduced since only half of it is calculated; however, there is a lack of understanding of how to maintain the performance. The CSPNet paper also showed that the amount of computation was reduced through various experiments. However, the proof related to the maintenance of the performance could not be confirmed. 

### 2.2. Receptive Field

The semantic segmentation method classifies the class by approaching each pixel differently from object detection, which is detected in the form of a rectangular box when a class-categorized object is detected. Since the class is classified by approaching in pixel units, it has the advantage of being able to distinguish the class more precisely than detection in the form of a rectangular box. However, high-performance hardware is required for autonomous vehicles that require real-time performance, as it requires substantial computation. Therefore, the semantic segmentation method is being extensively conducted on performance and convolution techniques that can reduce the amount of computation. Among them, many studies using the receptive field have been conducted.

The receptive field can be thought of as the size of the kernel viewed at a time during convolution. Therefore, if the receptive field is large, a large number of areas can be calculated at once, improving the overall division performance. However, the accuracy of detecting the object to be distinguished is low, and, recently, a 3 × 3-sized kernel was almost fixed, and the convolution calculation was performed. Therefore, dilated convolution [10], which can secure a larger receptive field with the same amount of computation, and depth-wise separable convolution [11], which divides channel information and spatial information and merges the output values into one, are convolution techniques that can reduce typical amounts of computation. 

### 2.3. Attention

Attention, used in the algorithm proposed in this paper, is an algorithm derived from LSTM. The vanishing gradient problem, which is the most prominent problem in RNN, occurred, and information loss occurred when all information was compressed into a vector in a single fixed size. Therefore, in order to solve the problem, i.e., the accuracy of the output sequence decreases when the input sequence becomes longer, the encoder emphasizes the part of the input word related to the word to be predicted at the corresponding point in the entire input sentence in the encoder each time the decoder predicts the output word. Importantly, attention was able to perform this task.

Attention—in image processing—has received little attention. Attention in image processing has been used substantially in the sequence structure to find the context that the model should focus on during the process or in the video. However, with the advent of self-attention [12], the concept of attention has been expanded. [13] It was used to refer to focusing on a specific part and began to be applied to traditional image algorithms. Even in the structure proposed in this paper [14], the performance of the algorithm is improved by using channel attention and spatial attention [15] at the end of one block.

## 3. Calibration

### 3.1. Camera Calibration

To develop an algorithm using a camera, the first step is to correct camera distortion. If the algorithm is developed without correcting the distortion, the accuracy is lowered because a distorted object is detected. Due to the nature of the camera lens, distortion is unconditionally generated. First, to correct this, there are two types of distortion: (1) radial distortion and (2) tangential distortion. First, radial distortion is caused by the refractive index of the convex lens. The distortion originates from the distance from the center. Tangential distortion is distortion that occurs in the process of assembling the camera because the image sensor and the camera lens are not horizontal or the center point is not aligned. Therefore, a process known as camera calibration obtains the position where points in 3D are projected through a lens, and reconstructs the 3D spatial coordinates from the image coordinates.

As can be seen in the camera coordinate system, the feature points in the three-dimensional space are projected onto the two-dimensional image plane.

(1) $$s\left[\begin{array}{c} x\\ y\\ 1 \end{array}\right]=\left[\begin{array}{ccc} f_{x} & skew_{cf_{x}} & c_{x}\\ 0 & f_{y} & c_{y}\\ 0 & 0 & 1 \end{array}\right]\left[\begin{array}{cccc} r_{11} & r_{12} & r_{13} & t_{1}\\ r_{21} & r_{22} & r_{23} & t_{2}\\ r_{31} & r_{32} & r_{33} & t_{3} \end{array}\right]\left[\begin{array}{c} X\\ Y\\ Z\\ 1 \end{array}\right]=A[R|t]\left[\begin{array}{c} X\\ Y\\ Z\\ 1 \end{array}\right]$$

Equation (1) is the pinhole camera transformation model. By looking at Equation (1), it is converted into the pixel coordinate system projected onto the image plane through ***A*** and [R|t] in the 3D world coordinate system, where ***A*** is an intrinsic parameter and [R|t] is an extrinsic parameter. Since the internal parameters refer to the internal parameters of the camera, such as the focal length and center point of the camera, calibration was performed only once during the experiment. In contrast, since the external parameters vary depending on the environment, such as the height and direction of the camera, and since the experiment was conducted in an environment without a tolerance correction room, the external parameters were corrected whenever the environment changed.

The camera’s internal parameters include the focal length, main point, and asymmetry coefficient. Here, the asymmetry coefficient indicates the degree of inclination of the image sensor. Since modern cameras do not consider the asymmetry coefficient, only the focal length and the main point need to be corrected. The focal length is the distance between the camera lens and the image sensor. Among the parameters corresponding to ***A*** in Equation (1), it corresponds to $f_{x}$ and $f_{y}$. If the focal length of the lens is short, the angle of view becomes larger as the size of the object becomes smaller with a wide-angle lens. Among the parameters corresponding to ***A*** in Equation (1), $c_{x}$ and $c_{y}$ are the main points and denote the center of the camera lens. Here, the center of the lens is given by the coordinates of the foot of the water line that is lowered from the pinhole to the image sensor.

In Equation (1), s is the scale value resulting from the influence of homogeneous coordinates. The external parameter corresponding to [R|t] refers to a transformation relationship that occurs through rotational transformation and translation between the camera coordinate system and the world coordinate system. In the case of an external parameter, it is not a parameter that the camera intrinsically has; therefore, external parameters depend on the direction or height. In this study, the external parameters were corrected by visually changing the roll, pitch, and yaw through the four images—before, after, left, and right—after the internal parameters were corrected.

### 3.2. Bird Eye View Image Registration

**Figure 1:**
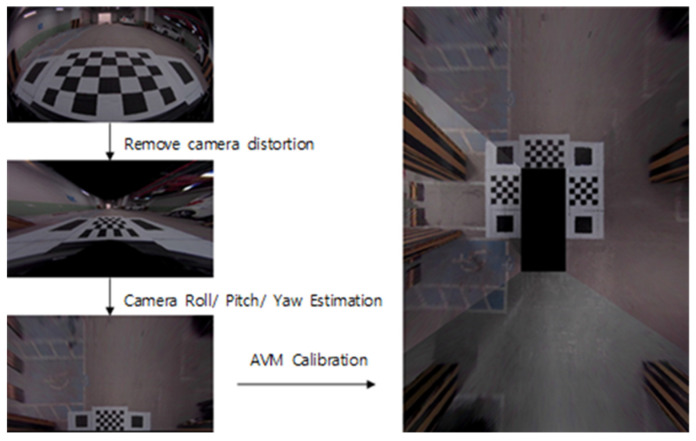
AVM calibration and registration.

To produce AVM images, calibration was performed using four Sekonix 190° wide-angle cameras. As shown in Figure 1, the distortion of the image received from the four wide-angle cameras is removed. Since a wide-angle view camera is designed with a wide-angle lens, it is difficult to model from a general perspective. A fisheye lens system with distortion removal [16] and a linear distance was used. Fisheye transparency is used to find the distortion point, and the final pixel coordinate vector u, v is obtained to remove the distortion. A single AVM calibration image was created through registration.

## 4. Proposed Area Detection Algorithm

### 4.1. Network Structure of Semantic Segmentation 

In this paper, the HarDNet algorithm with a good performance and low computational requirements was used to detect the parking area and collision risk area. Although the amount of computation was greatly reduced, substantial computation was required in the network structure to secure a real-time performance in autonomous parking situations. CSPNet was applied to HarDNet. As in the existing algorithm, when the size of the feature map channel is reduced by 1/2, the performance is reduced by more than 5%, and the amount of computation is reduced by setting the size of the feature map channel to 2/3, which experimentally minimizes the performance degradation.

**Figure 2:**
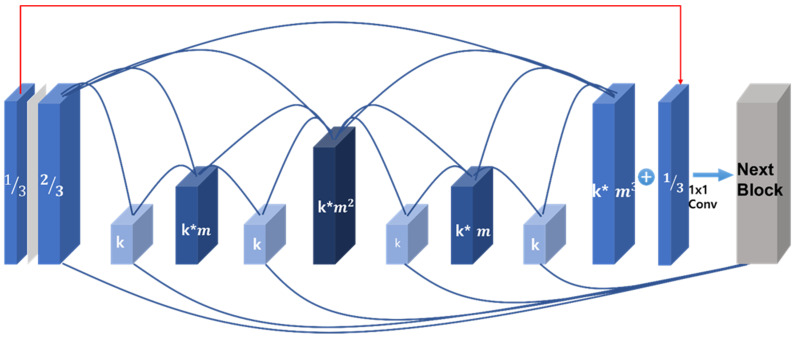
The proposed CSPHarDNet structure.

In Figure 2, k is convolution channel, m  is the channel weight, and the output structure of CSPHarDNet [17], proposed in this paper, is shown. Based on the fusion first method of CSPNet, the input of the feature map in the figure is divided into 2/3- and 1/3-sized channels; the 2/3 channel performs the convolution operation with HarDNet, and the 1/3 channel is the last feature that has been calculated. After performing 11 convolutions with the map and channel sum, the feature map is output as the next block by summing the channels with the feature maps of odd-numbered layers.

The structure of the decoder is the same as the decoder used in HarDNet, and the size of the input image is enlarged by using bilinear interpolation [18], which enlarges the image size by N times.

**Figure 3:**
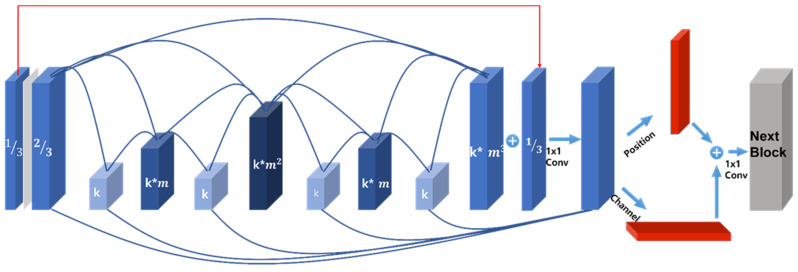
The proposed final attention HardNet structure.

Figure 3 is an architectural structure using attention to improve the performance in CSPHarDNet. In order to improve the performance of the class, in this study, a channel attention block using MLP and pooling and a position attention block using MAX and pooling are created and applied to the feature map before moving on to the next block after the CSPHarDNet block is completed and passed to the next block.

**Algorithm 1** The proposed final attention HardNet1.     **Input:** input channel2.     **Output:** output channel O
3.     Channel layer k, weight value
m
4.     **For** HardNet block do
5.     **If**
k is odd number then
a.            
k
 × m
6.       **end**
7.       **If**
k is even number then
a.            
k × (m×n)8.       **end**
9.     **end**
10.   O = {$odd\;number\;k_{1}$ + …$odd\;number\;k_{n}$}
11.   Channel attention = MLP(Avgpool(O) + MLP(Maxpool(O)
12.   Position attention = $f^{7\times 7}$(Avgpool(O)Maxpool(O))
13.   O = channel attention + Position attention


As can be seen in the pseudocode above, the proposed algorithm maintains the minimum computational complexity by creating a final feature map with the sum of channels with a small channel size, and improves the accuracy by channel attention using MLP, and position attention using convolution.

### 4.2. Network Learning Methods

In order to learn the proposed network, experiments were conducted while various activation functions, loss functions, and optimization techniques were adjusted. Furthermore, the algorithm that showed the best performance will be explained.

During learning, an input is received and passed through a nonlinear function before being passed to the next layer, and the function used at this time is known as an activation function. Recently, most activation functions use ReLU, which has a value of 0 for negative numbers and infinite values for positive numbers; however, the proposed network uses Mish [19], which was published in 2019. The positive part of Mish, which can be expressed as Equation (2), extends to infinity (similar to ReLU) to avoid saturation, and the negative part allows for some negative non-zero values to emphasize the slope value of the function.

(2) $$f\left(x\right)=x\tanh\left(softpuls\left(x\right)\right)=x\tanh(\ln\left(1+e^{x}\right))$$

The loss function is an indicator of the learning state and represents the difference between the desired output value and the model output value. Learning is the process of finding weights and biases that minimize the loss function. A common loss function is cross-entropy.

(3) $$Cost=-\overset{C}{\underset{k=1}{\sum }}t_{k}\log\left(y_{k}\right)$$

The cross-entropy error can be expressed as Equation (4). The $t_{k}$ value is a one-hot encoded vector, and the output value $y_{k}$ is multiplied by the natural logarithm. Assuming that the correct answer is 1, the error converges to 0 as it approaches the correct answer. Conversely, as the distance from the correct answer increases, the error increases, and a greater penalty is given as the distance from the correct answer is increased through the cross-entropy error.

(4) $$Cost=-\overset{C}{\underset{k=1}{\sum }}{\left(1-y_{k}\right)}^{\gamma }t_{k}\log\left(y_{k}\right)$$

Focal loss [20] is used in the proposed network. Focal loss adds ${\left(1-y_{k}\right)}^{\gamma }$ from the existing cross-entropy error to apply more weight to a difficult-to-classify problem than to an easy-to-classify problem, resulting in a good performance in object detection.

The optimization technique refers to a technique that minimizes the loss function through parameter updating. The most commonly used optimization techniques include stochastic gradient descent (SGD) and Adam (Adagrad + RMSProp). In the network proposed in this study, AdamW [21] was used. This included Equation (5), Equation (6), Equation (7), Equation (8), and AdamW’s formula. α is learning, β is the moment, and M(t) and V(t) are the exponential moving average.

(5) $$M\left(t\right)=\beta_{1}M\left(t-1\right)+\left(1-\beta_{1}\right)\frac{\partial }{\partial w\left(t\right)}\left(Cost\left(w\left(t\right)\right)+wx_{t-1}\right)$$

(6) $$V\left(t\right)=\beta_{2}V\left(t-1\right)+\left(1-\beta_{2}\right)\frac{\partial }{\partial w\left(i\right)}{\left(Cost\left(w\left(t\right)\right)+wx_{x-1}\right)}^{2}$$

(7) $$\hat{M}\left(t\right)=\frac{M\left(t\right)}{1-\beta_{1}^{t}}\;\;\;\;\hat{V}=\frac{V\left(t\right)}{1-\beta_{2}{}^{t}}\;\;$$

(8) $$W\left(t+1\right)=W\left(t\right)-\left(\alpha *\frac{\hat{M}\left(t\right)}{\sqrt{\hat{V\left(t\right)+\epsilon }}}+wx_{t-1}\right)$$

AdamW can be considered as adding weight decay to the existing Adam. By observing the equation, AdamW’s formula is the same as Equations (5), (6), (7), and (8). Equations (5) and (6) are estimates of the moment of the gradient, respectively, of the exponential mean and the mean of square values. Equation (7) is used for bias correction for the two equations, and Equation (8) updates *W*, a weight, by initializing *M* and *V* to 0. For the hyperparameters used in the formula, α = 0.001, $\beta_{1}$ = 0.9, $\beta_{2}$ = 0.999, and ϵ = $10^{-8}$ were applied. Unlike Adam, $wx_{t-1}$ is added for the weight update so that the weight decay effect can be seen.

### 4.3. Data Augmentation and Network Optimization

**Figure 4:**
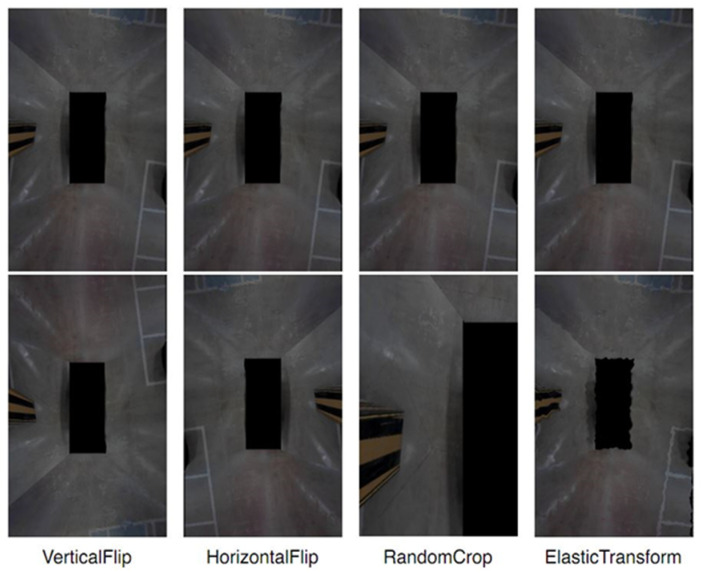
Data augmentation methods for training.

Data augmentation [22] is used to artificially change the image to the extent that it can be used for learning. In this study, four types of data augmentation were used, as shown in Figure 4. VerticalFlip and HorizontalFlip are techniques that reverse the top and bottom and left and right of the image; RandomCrop is a technique that enlarges only a part of the image; ElasticTransfrom is a technique that gives elastic noise to the image. Inverting or adding noise to the image helps to prevent overfitting and helps generalization learning; however, in this study, the amount of data was insufficient because the data were directly created. Therefore, in order to collect sufficient data, data augmentation was used to add data.

Since autonomous driving requires real-time image processing according to the moving speed of the vehicle, the speed of the algorithm executed in the embedded system is important. Therefore, network optimization is essential for deep learning in fields where real-time image processing is important. First, automatic mixed precision (AMP) [23], which can efficiently use GPU in the deep learning process, is a learning method using floating-point arithmetic.

In the floating-point method, there is 1 bit, indicating the sign at the beginning, and, in the case of Float32 (single precision), the 8-bit exponent and 23-bit mantissa are stored; in the case of Float16 (half precision), the 5-bit exponent and 10-bit mantissa are stored. If Float32 is used, calculation takes a long time since the number of bits used for the operation is high; however, the precision is high. Conversely, if Float16 is used, the calculation time is short, but the precision is lowered. Currently, Float32 is used for deep learning. If operating with Float16, storage space can be saved and the operation can be accelerated. However, since the precision is low, AMP is an algorithm that uses Float32 and Float16 together to increase speed and maintain precision.

**Figure 5:**
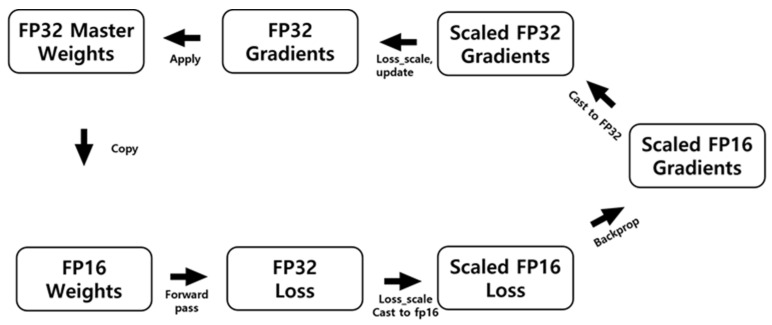
AMP training process.

The operation method of mixed precision when learning the network is shown in Figure 5. First, to use FP16 for forward and backward operations, I create an FP16 copy weight for the FP32 weight. Then, a forward pass is performed using the FP16 copy weight. I cast the predicted value of FP16 calculated by the forward pass to FP32. The FP32 loss is calculated through the FP32 prediction value and multiplied by the scaling factor. The scales the FP32 loss to FP16 and then backwards using the scaled FP16 loss, and the gradient is calculated. Finally, I cast the FP16 gradient to FP32, divide it by the scaling factor, and update the FP32 weight with the FP32 gradient. If this is carried out, the FP32 weight is continuously stored, and, since the FP16 copy weight performs forward and backward operations, it is possible to maintain precision and reduce memory.

After reducing the training time and memory by using AMP, the trained model is ported to an actual embedded device. When porting to embedded devices, models can be optimized, and TensorRT is a function used in this case. TensorRT improves the inference speed on NVIDIA GPUs by optimizing models trained with NVIDIA-provided APIs. Due to the fact that TensorRT can be used as an API, it can be used without learning CUDA.

TensorRT, like AMP, lowers precision in order to speed up inference. In the case of TensorRT, the precision of FP32 data can be reduced to FP16 and INT8 data types. In this study, the model accuracy was not affected since the experiment was conducted by lowering it to FP16; however, additional calibration is required to lower the precision to the INT8 data type.

**Figure 6:**
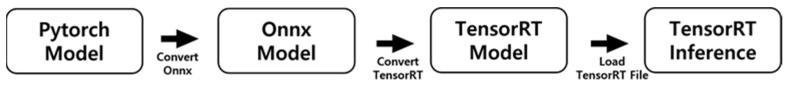
TensorRT conversion process.

In order to infer using TensorRT, there are four simple steps, as shown in Figure 6. Since Pytorch is used in this paper, the trained model is defined as the PyTorch model. First, in order to use C++-based TensorRT, the PyTorch model learned based on Python is converted through Onnx. This converted Onnx file can be converted into a TensorRT file, and inference can be made in TensorRT through the converted TensorRT model.

## 5. Experiment

### 5.1. Experimental Environment and Evaluation Method

Table 1 shows the composition of the training dataset and validation dataset created for network training and validation. Four hundred training data points, 100 validation data points, and a total of 500 AVM images and label images were created. Due to the fact that the 400 training data points were too small to reliably verify the performance of the model, learning was conducted with a total of 1600 images through data augmentation.

In order to accurately verify the model based on the learned model, the National Information Society Agency DB was additionally used. NIA DB is a parking scenario DB created by NIA. The front dataset consists of a total of 20 objects with 120 images of 1920 × 1080 size, and the AVM dataset was created with 120 images consisting of a total of 7 objects with 1280 × 720 images. For performance verification, 1470 AVM images of 1280 × 720 size were used for verification.

**Figure 7:**
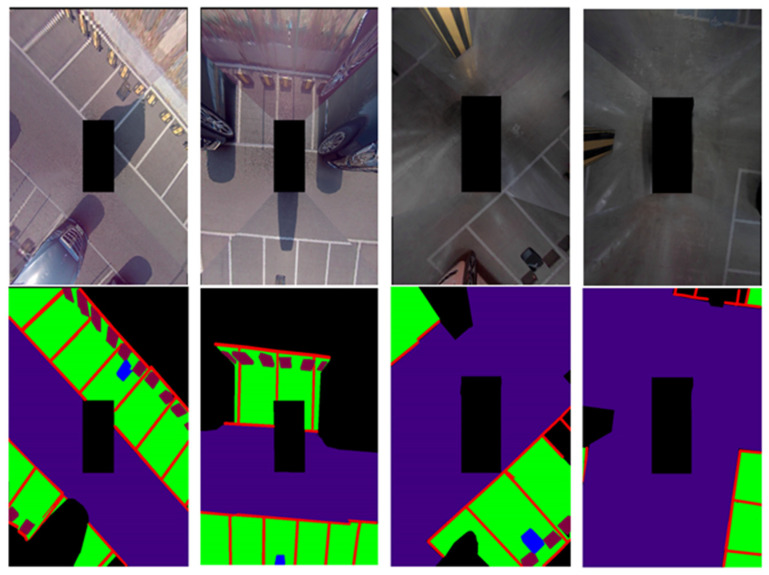
Examples of data labeling.

Figure 7 shows an example photo of labeling data. The first row is the training image, and the second row is the labeled ground truth image. In the ground truth image, red is the parking line, green is the parking area, and blue is the wireless charging pad not used in this study. Purple refers to the driving area and light purple refers to the stopper. In the ground truth image, the parking area is an area where a vehicle can be parked without collision, and the unlabeled area is defined as a collision risk area.

For the training image size, the original AVM image of 1,024,939 was used as-is; however, since the network is large and the image size is large, four GPUs were used for training. The AMP test was conducted only on a high-performance PC (Table 2). The GPU used in the experiment was a GeForce RTX 3090. Moreover, the AMP algorithm was used, and the learning speed and memory usage were compared to those when the experiment was conducted. 

**Figure 8:**
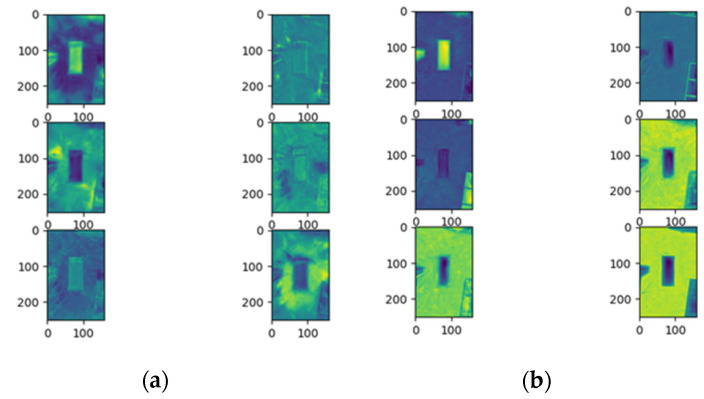
Comparison of feature maps by Epoch: (**a**), Epoch 1; (**b**), Epoch 400.

In order to assess the performance of attention in the CSPHarDNet architecture proposed in this paper, I visualized the feature map of position attention and channel attention added to each block using the hook function provided by PyTorch. The hook function can visualize the feature map result of the desired layer during training. Figure 8 is a visualization of the feature map of the final layer. In the initial Epoch 1, it is impossible to evaluate which part was learned since there was minimal learning; however, in Epoch 400, it can be visually confirmed that the six objects to be detected are learned, as learning is in progress.

The final test was conducted on a NVIDIA Jetson Xavier, and the proposed network—based on the ImageNet Pretraining model in Table 3—was trained on four GPUs, and a 4GPU model was created. Moreover, a 1GPU model was trained on 1 GPU based on the pre-trained 4GPU model, creating the final model. Afterwards, Onnx was used to change to a C++-based model, converted into a TensorRT file, and a model that can be inferred from TensorRT was created and tested on an NVIDIA Jetson Xavier. Lastly, for verifying the proposed network accuracy, IoU (intersection over union)—the most used metric in the semantic segmentation field—was used.

### 5.2. Experimental Results

**Figure 9:**
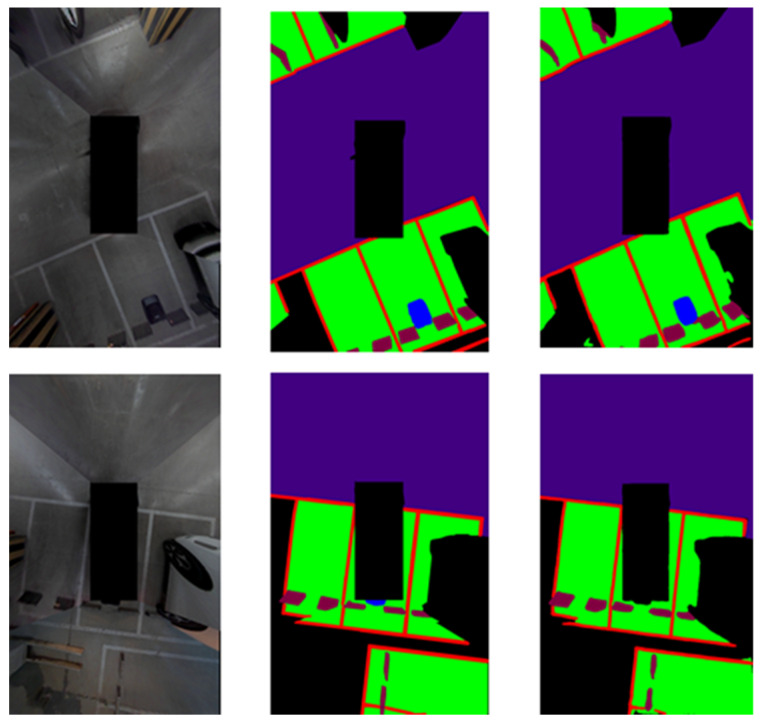
Indoor semantic segmentation detection results. Left: input; middle: ground truth; right: output.

**Figure 10:**
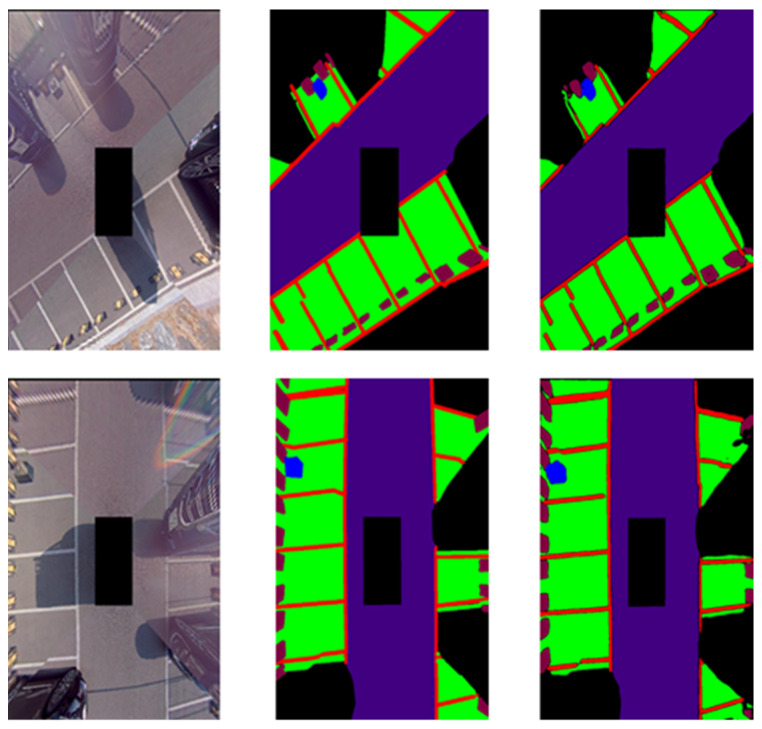
Outdoor semantic segmentation detection results. Left: input; middle: ground truth; right: output.

In order to demonstrate the experimental results, learning was carried out with the proposed network using the training data of 400 AVM images acquired from the parking lot of Chungbuk National University. The obtained learning data and evaluation data were acquired at the same location; however, data acquired at different points in time were used for the learning data and evaluation data. Figure 9 and Figure 10 show the results of the experiment indoors and outdoors, respectively. The first image is the original image, the second is the ground truth, and the third is the verification result. This study was carried out to obtain results from a parking situation, and only the learning data in the parking situation were learned; the experimental results were obtained by acquiring the verification data in a similar environment. Therefore, it can be confirmed that the whole is consistent except for the error originating from the boundary part of the object.

Table 3 shows the results for the IoU and detection speed based on the validation data using the attention CSPArDNet model. The Python model—created on the deep learning PC—was changed to a C++ model and run with a similar performance in Xavier, and the mIoU of the final evaluation table in Xavier shows the performance of the attention HarDNet result.

Table 4 shows the results of the performance analysis conducted by the National Information Society Agency to demonstrate the performance of HarDNet and CSPHarDNet object detection proposed in this paper. With the existing HarDNet network, 83.31% of mIoU and 15.44 FPS were obtained. The CSPDenseNet—used to increase the detection speed—obtained 79.84% mIoU and 19.98 FPS, and the CSPHarDNet proposed in this paper obtained 81.89% mIoU and 18.15 FPS. Since DenseNet has more overlapping weights than HarDNet, CSPDenseNet showed higher FPS than the experimental result. In contrast, CSPHarDNet did not have more overlapping weights than DenseNet in the HarDNet structure; therefore, only 1/3, not half, of the channels were used in the fusion-first method to create the CSPHarDNet structure, resulting in a slight increase in FPS and higher mIoU than CSPDenseNet. If I simply compare one billion FLOPs (BFLOPs), CSPDenseNet provided the best results. However, this study proposed the detection of parking areas and collision risk areas in a 10 km/s parking scenario, and CSPHarDNet was more suitable as a result of conducting experiments with various obstacles using cars, people, and parking cones during the experiment for the generalization of the model. The network-based algorithm proposed in this study obtained lower mIoU than the HarDNet semantic segmentation algorithm; however, since the detection speed was increased by an average of 3 FPS, the experiment was conducted regardless of the real-time parking situation.

**Figure 11:**
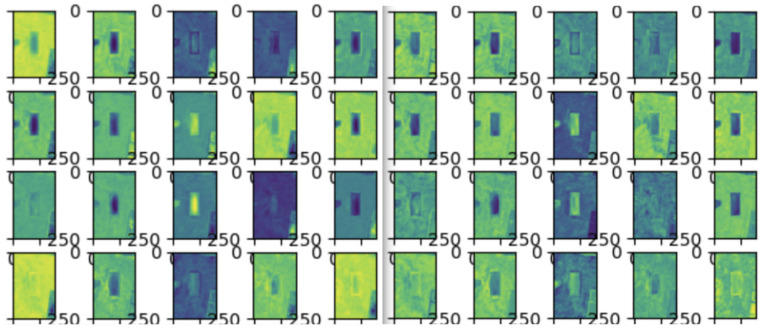
Visualization of attention feature map. Left: channel attention; right: position attention.

Figure 11 shows the result of the attention experiment using PyTorch’s hook, and only 20 channels from the selected feature map were visualized after attention was progressed and before moving on to the next layer. First, the channel attention part was studied for the purpose of improving the perception of the parking line, parking space, collision risk area, drivable area, stopper, and charging pad, which were the targets to be detected. It can be seen that a great deal has been learned. However, in the case of position attention, since object-oriented learning is taught as a whole again, the learning result for the overall division—rather than the attention of a specific object—can be seen.

**Figure 12:**
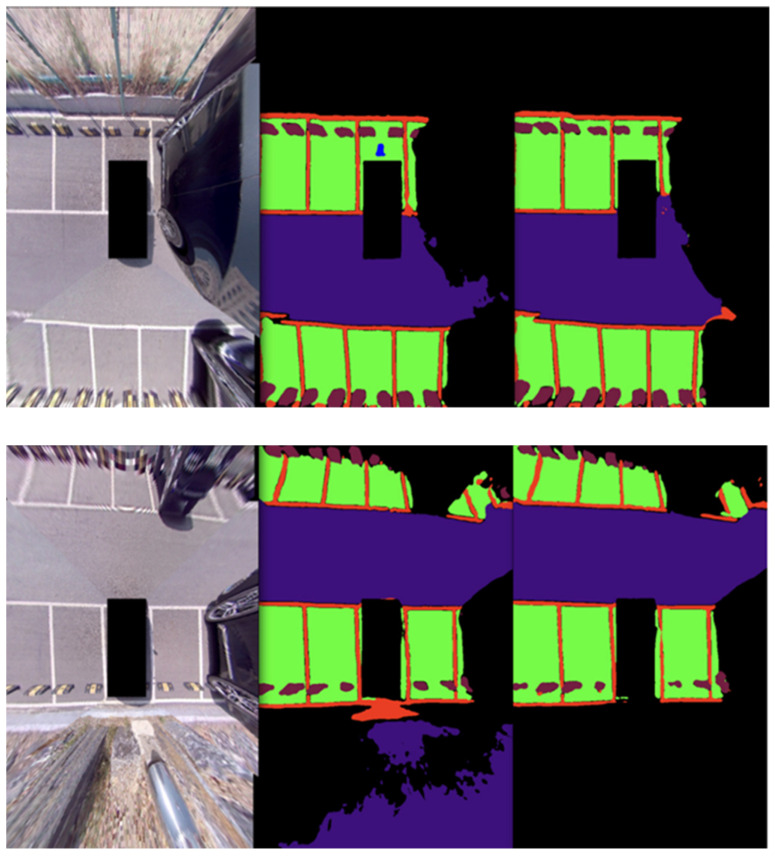
Performance comparison of attention. Left: input; middle: CSPHarDNet; right: attention.

Figure 12 shows an image comparing the performance of HarDNet and HarDNet with attention added. First, in the case of HarDNet, there was an error in recognizing the charging pad behind the vehicle and an error in recognizing the collision risk area as a drivable area. Thus, I solved the problem by adding attention to the existing HarDNet to increase the algorithm performance.

Two optimization experiments were carried out. First, Table 5 shows the performance comparison of AMP. Performance experiments were conducted based on attention CSPHarDNet. GeForce RTX 1080 and GeForce RTX 3090 were the two GPUs used when AMP was and was not used, respectively. Moreover, the accuracy, memory usage, and learning time were compared. When using AMP based on GeForce RTX 1080, the performance and learning time did not decrease, and the memory decreased by approximately 30%. When the experiment was conducted with GeForce RTX 3090, the performance did not decrease, the memory decreased by 30%, and the learning time decreased by 30%. Both GPUs reduced memory without reducing the performance. However, since PyTorch AMP supports a function to reduce the learning time from GeForce RTX 2080 or later, the learning time was different.

Table 6 shows the performance comparison of TensorRT. In the case of TensorRT, the mIoU and detection speed of the existing HarDNet, CSPHarDNet, and CSPHarDNet+ attention proposed in this paper were compared. The accuracy of all three models did not decrease, and it was confirmed that the detection speed was approximately twice as fast as the NVIDIA Xavier standard.

**Figure 13:**
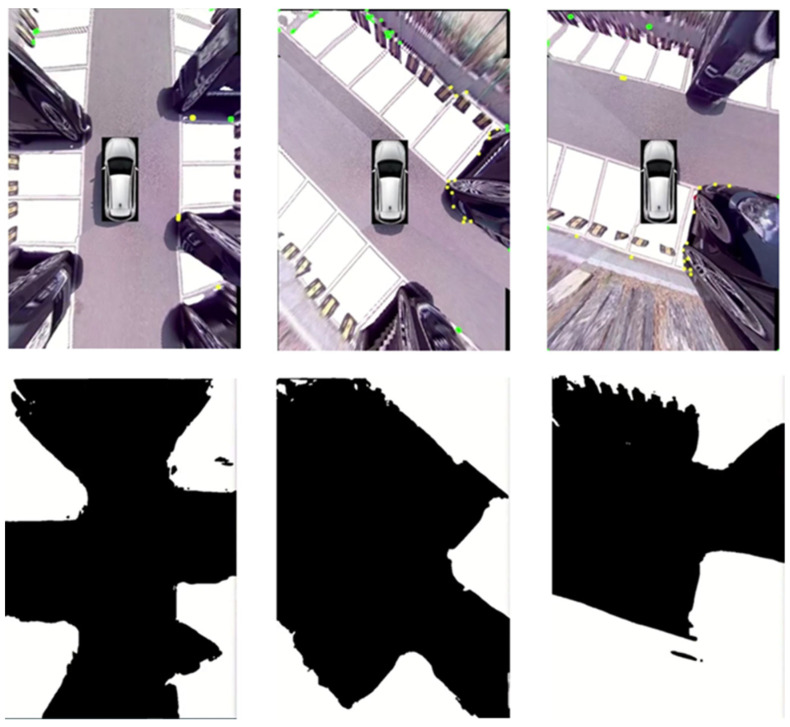
NVIDIA Xavier detection results.

Figure 13 shows results of the final test, which was conducted in the NVIDIA Xavier environment, and the experiment was conducted based on attention CSPHarDNet, which demonstrated the best performance and is suggested in this paper. In the parking situation, the result of the parking part is detected in row 1 (white), and the result of detecting the area not detected in the semantic segmentation result is in the second row (white). When evaluating the objects detected as collision risk areas in row 2, stationary cars, walls, flower beds, etc., were detected.

## 6. Conclusions

In this paper, research was conducted in order to detect parking areas and collision risk areas based on deep learning using a camera sensor. Importantly, I created a dataset in the parking lot of Chungbuk National University in order to detect it from an AVM image. A network was constructed using the semantic segmentation algorithm, and CSPNet and attention were added to implement an optimal algorithm that could be used with good performance in a real-world vehicle environment. Various attempts were made for the activation function, optimization technique, and loss function to find the optimal conditions and learning. In order to use the original images as-is, an encoder was constructed, considering the amount of computation. For optimizing the training network, we used AMP to reduce the training memory and training time and used TensorRT in order to achieve an over two-fold FPS. By converting the desktop (PC)-created model to C++, we found that it was possible to detect the parking area and the drivable area with the NVIDIA Xavier and to detect the other areas as collision risk areas in order to detect the parking area and the collision risk area in indoor and outdoor environments. For the performance test, the self-produced AVM image and the image received from the National Institute of Intelligent Information Society (NIA) were tested, and an actual vehicle test was conducted in the Chungbuk National University parking lot. In future work, our results would be suitable in an environment similar to the currently learned data; however, the results were not obtained in new environments, e.g., parking lots with illegal parking, women-only parking lots, and parking lots for the disabled. For future work, I plan to conduct research for improving the performance. The final goal of this study is to detect collision-risk objects while parking and, to measure the exact distance between objects and vehicles, I plan to implement an algorithm to find the distance based on a model learned using a camera and LiDAR. I plan to conduct research to find the exact location of the detected object using only the camera in a real vehicle.

## Figures and Tables

**Table 1:** AVM image-based parking area—travelable area dataset.

Image	Training Data	Test Data
AVM Image	1600	100
Label Image	1600	100

**Table 2:** High-performance PC specifications.

CPU	Intel Core i9-10900x CPU 3.70 GHz
RAM	128 GB
OS	Ubuntu 18.04LTS
GPU	GeForce RTX 3090 × 4

**Table 3:** Object detection performance and speed evaluation table.

Model	mIoU(%)	FPS(PC)	FPS(Xavier)	Cross EntropymIoU(%)	Focal Loss mIoU(%)	ReLU mIoU(%)	Mish mIoU(%)
Attention CSPHarDNet	83.88	17.33	17.45	83.24	83.79	83.01	83.79

**Table 4:** NIA dataset results.

Model	mIoU(%)	BFLOPS	FPS(PC)	FPS(Xavier)
HarDNet	83.31	110.39	15.44	15.92
CSPDenseNet	79.84	60.356	19.98	20.11
CSPHarDNet	81.89	72.847	18.15	18.36
Attention CSPHarDNet	83.76	81.443	17.33	17.45

**Table 5:** AMP performance comparison.

GPU	AMP	Accuracy (%)	Memory (MB)	Training Time (Hour)
GeForce GTX 1080	X	84.15	10423	6
GeForce GTX 1080	O	84.21	6854	6
GeForce RTX 3090	X	84.17	10788	6
GeForce RTX 3090	O	84.22	6932	4

**Table 6:** TensorRT performance comparison.

Model	TensorRT	mIoU(%)	FPS
HarDNet	X	80.31	15.92
HarDNet	O	80.12	32.89
CSPHarDNet	X	79.84	20.11
CSPHarDNet	O	79.22	41.46
Attention CSPHarDNet	X	82.89	18.31
Attention CSPHarDNet	O	82.57	37.41

## Data Availability

Not applicable.
